# Clinical Implication of Coronary Tortuosity in Patients with Coronary Artery Disease

**DOI:** 10.1371/journal.pone.0024232

**Published:** 2011-08-31

**Authors:** Yang Li, Chengxing Shen, Yanan Ji, Yi Feng, Genshan Ma, Naifeng Liu

**Affiliations:** Department of Cardiology, Zhongda Hospital, Southeast University, Nanjing, China; University of Modena and Reggio Emilia, Italy

## Abstract

**Background:**

Coronary tortuosity (CT) is a common coronary angiography finding. The exact pathogenesis, clinical implication and long-term prognosis of CT are not fully understood. The purpose of this study is to investigate the clinical characteristics of CT in patients with suspected coronary artery disease(CAD) in a Chinese population.

**Methods:**

A total of 1010 consecutive patients underwent coronary angiography with complaints of chest pain or related symptoms were included in the present study (544 male, mean age: 64±11 years). CT was defined by the finding of ≥3 bends (defined as ≥45° change in vessel direction) along main trunk of at least one artery in systole and in diastole. Patients with or without CAD were further divided into CT-positive and CT-negative groups, all patients were followed up for the incidence of major adverse cardiovascular events (MACE) for 2 to 4 years.

**Results:**

The prevalence of CT was 39.1% in this patient cohort and incidence of CT was significantly higher in female patients than that in male patients (OR = 2.603, 95%CI 1.897, 3.607, P<0.001). CT was positively correlated with essential hypertension (OR = 1.533, 95%CI 1.131, 2.076, P = 0.006) and negatively correlated with CAD (OR = 0.755, 95%CI 0.574, 0.994, P = 0.045). MACE during follow up was similar between CAD patients with or without CT.

**Conclusions:**

CT is more often seen in females and positively correlated with hypertension and negatively correlated with coronary atherosclerosis.

## Introduction

Coronary artery disease (CAD) is the leading cause for mortality and morbidity severely affecting the quality of life in both developed and developing countries worldwide [1], [2]. Coronary angiography is still the golden standard for the diagnosis of CAD and coronary tortuosity (CT) is a common coronary angiography(CAG) finding. Previous studies suggested that CT might be associated with chronic pressure load and impaired left ventricular relaxation and possibly coronary ischemia [3]–[5]. However, the impact of CT on prognosis in CAD patients remains largely unknown. In this study, we observed the prevalence of CT in a large cohort patient population underwent coronary angiography due to chest pain and similar symptoms and explored the association between CT and incidence of coronary stenosis, moreover, the impact of CT on prognosis was determined in patients with and without CAD.

## Methods

### Subjects

A total of 1010 consecutive patients underwent coronary angiography due to chest pain and similar symptoms were included in the present study (544 male, mean age: 64±11 years).

**Figure 1:**
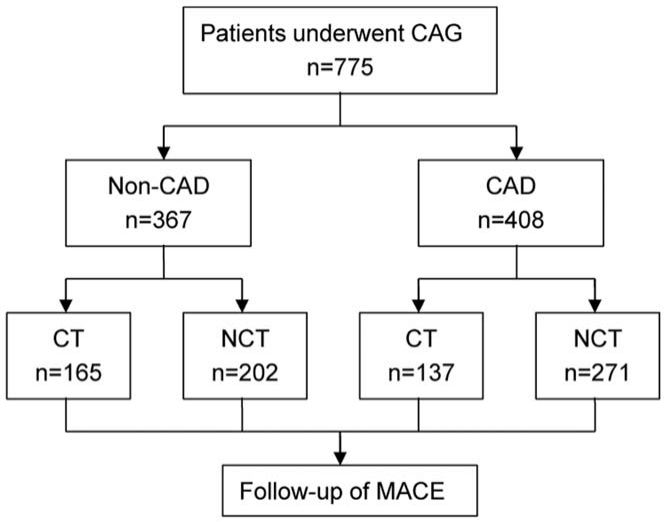
Flow diagram of patient follow-up.

All patients were followed up for 2 to 4 years post coronary angiography. Patients were divided into 4 groups according to the presence and absence of CAD and CT (Figure 1). The study was approved by the hospital ethics committee (Zhongda Hospital, Southeast University) and in accordance with the principles embodied in the Declaration of Helsinki, written informed consent was obtained from each participating patient.

### Risk factors

Essential hypertension was defined as systolic blood pressure of ≥140 mmHg or diastolic blood pressure of ≥90 mmHg, or taking antihypertensive medication. Hyperlipidemia was diagnosed with total plasma cholesterol level of ≥200 mg/dl or low-density lipoprotein-cholesterol level of ≥130 mg/dl or triglyceride level of ≥150 mg/dl, or taking cholesterol-lowering drugs. Diabetes mellitus was defined by WHO criteria. Heart function was evaluated based on ACCF/AHA 2009 heart failure guideline [6], stage C or D was identified as chronic heart failure. Smokers were defined as those smoking regularly.

### Coronary angiography

**Figure 2:**
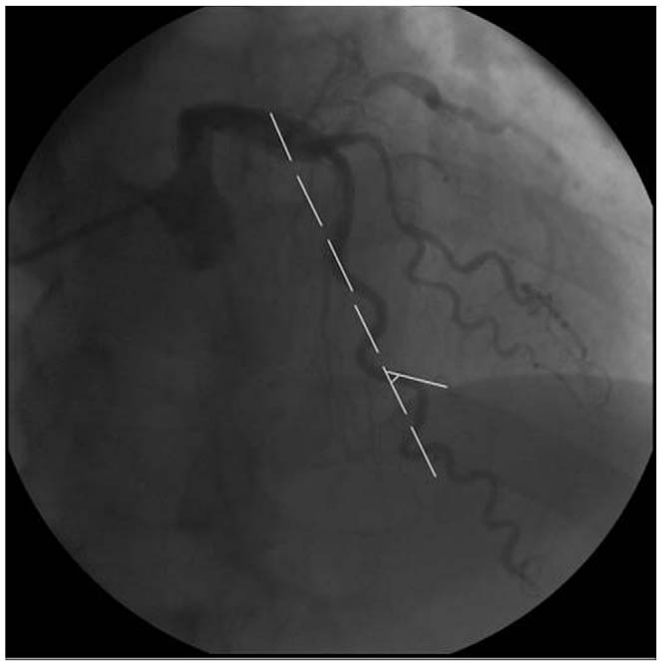
Diagnosis of coronary tortuosity.

All patients underwent elective coronary angiography according to the Judkins technique using a Philip FD-10 X-ray system. The left anterior descending coronary artery (LAD), the left circumflex coronary artery (LCX) and the right coronary artery (RCA) were observed by various angulations. Images were recorded on CD-R. CAD was verified as ≥50% luminal narrowing in at least one main coronary artery. CT was evaluated on special angulations, LAD was assessed in right anterior oblique with cranial angulations and LCX in left anterior oblique with caudal angulations, while RCA in right anterior oblique. CT was identified by ≥3 bends (defined as ≥45° change in vessel direction) along main trunk of at least one artery, present both in systole and in diastole [4] (Figure 2).

### Major adverse cardiovascular events (MACE)

Cardiac death was defined as death of cardiac causes or any death without another known cause. Acute myocardial infarction was defined in accordance with the universal definition proposed [7]. Coronary revascularization was defined as angioplasty or stenting or coronary artery bypass grafting. Stroke was defined as loss of neurologic function due to an ischemic or hemorrhagic event. Composite end point included all cause mortality, myocardial infarction, coronary revascularization, non-fatal angina needing hospital admission and stroke. MACE was verified by hospital medical records and telephone.

### Statistical Analysis

The data were analyzed using the statistical software package of SPSS (SPSS Inc., Chicago, IL, USA, Version 13). Continuous variables were expressed as mean ± standard deviation and categorical variables as percentages. Continuous variables between groups were compared by unpaired Student's t test. Categorical variables were compared by Chi-square test. Multivariate logistic regression analysis was performed, odds ratio (OR) and 95% confidence intervals (CI) were calculated. Two-tailed P values<0. 05 were considered significant.

## Results

### Prevalence of coronary tortuosity

The prevalence of CT was 39.1% in this cohort. Incidence of CT was significantly higher in the females than in males (62.8 vs. 37.2%, P<0.001) and in non CAD patients than in CAD patients (45.1% vs. 34.4%, P = 0. 001). Among the three coronary arteries, incidence of CT was 26. 9% in LCX, 21. 1% in LAD and 1.4% in RCA. CT was identified in both LAD & LCX in 9.9% of patients.

### Clinical characteristics of patients with or without coronary tortuosity

Of the 1010 patients, there were 723 patients with essential hypertension (71.6%, 381 male), 212 patients with diabetes mellitus (21%, 109 male), 198 patients with hyperlipidemia (19.6%, 87 male), 297 smokers (29.4%, 292 male), 170 patients with heart failure (16.8%, 99 male) and 569 patients with CAD (56.3%, 344 male).

Table 1 summarized the clinical characteristics of patients with suspected CAD with or without CT. CT incidence was significantly higher in females, hypertensive patients, cigarette smoker and patients without CAD.

**Table 1:** Clinical characteristics of coronary tortuosity.

Characteristic	CT group(n = 395)	NCT group(n = 615)	p value
age(year)	64±10. 3	64±10. 7	0. 690
female gender	247 (62. 8)	218 (35. 4)	<0. 001
hypertension	300 (75. 9)	423 (68. 8)	0. 014
diabetes mellitus	71 (18. 0)	141 (22. 9)	0. 059
hyperlipidemia	68 (17. 2)	130 (21. 1)	0. 125
cigarette smoking	72 (18. 2)	225 (36. 6)	<0. 001
chronic heart failure	64 (16. 2)	106 (17. 2)	0. 668
CAD	196 (49. 6)	373 (60. 7)	0. 001

Table 2 showed female gender and hypertension were associated higher incidence of CT while diabetes, hyperlipidemia and CAD were linked with reduced incidence of CT.

**Table 2:** Multivariate logistic regression analysis of selected variables for coronary tortuosity.

Variable	B	SE	*X* ^2^ value	P value	OR	95%CI
female	0. 957	0. 166	33. 083	<0. 001	2. 603	1. 897–3. 607
hypertension	0. 427	0. 155	7. 598	0. 006	1. 533	1. 131–2. 076
diabetes	−0. 353	0. 175	4. 158	0. 041	0. 702	0. 500–0. 986
hyperlipidemia	−0. 421	5. 787	5. 787	0. 016	0. 657	0. 466–0. 925
CAD	−0. 48	0. 140	4. 023	0. 045	0. 755	0. 574–0. 994

As shown in Table 3, female and hypertension were also related to higher incidence of LAD tortuosity while LAD atherosclerosis was negatively associated with LAD tortuosity. There were no significant difference between LCX(RCA) tortuosity and LCX(RCA) artherosclerosis.

**Table 3:** Multivariate logistic regression analysis of selected variables for LAD tortuosity.

Variable	B	SE	*X* ^2^ value	P value	OR	95%CI
female	1. 040	0. 165	39. 929	<0. 001	2. 830	2. 049–3. 907
hypertension	0. 612	0. 194	9. 963	0. 002	1. 844	1. 261–2. 697
LAD≥50% stenosis	−0. 192	0. 210	97. 095	<0. 001	0. 755	0. 574–0. 994

### Clinical characteristics and impact on prognosis of CT in patients with or without CAD

Table 4 showed the baseline characteristics of CAD patients with or without CT. There were significantly more females and hypertensive patients while less smokers and patients with three vessel disease in CT group than in NCT(no coronary tortuosity) group. There were more patients with three coronary vessel lesions in NCT group than in CT group.

**Table 4:** Baseline characteristics of the patients with CAD followed up.

Characteristic	CT group(n = 137)	NCT group(n = 271)	p value
age(year)	68±8.9	66±10.6	0. 163
female gender	74(54.0)	85(31.4)	<0. 001
medical history			
hypertention	112(81.8)	194(71.6)	0. 025
diabetes mellitus	32(23.4)	82(30.3)	0. 142
hyperlipemia	21(15.3)	59(21.8)	0. 122
cigarette smoking	33(24.1)	112(41.3)	0. 001
heart failure	29(21.2)	52(19.2)	0. 636
previous myocardial infarction	33(24.1)	68(25.1)	0. 824
previous coronary revascularization			
PCI	86(62. 8)	177(65.3)	0. 613
Coronary-artery bypass grafting	2(1.5)	3(1.1)	0. 760
No. of vessel (≥50% stenosis)			
One vessel	71(51.8)	119(43.9)	0.130
Two vessel	37(27.0)	64(23.6)	0.454
Three vessel	29(21.2)	88(32.5)	0.017
asprin therapy	115(83.9)	228(84.1)	0. 960
clopidogrel therapy	135(98.5)	267(98.5)	0. 990
statin therapy	75(54.7)	138(50.9)	0. 465
beta-blockers therapy	66(48.2)	127(46.9)	0. 802
calcium channel blocker	72(52.6)	136(50.2)	0.651
ACEI/ARB therapy	78(56.9)	142(52.4)	0. 385

Table 5 showed baseline characteristics of CT and NCT patients without CAD. There were significantly more females while less smokers in CT group than in NCT group.

**Table 5:** Baseline characteristics of the patients without CAD followed up.

Characteristic	CT group(n = 165)	NCT group(n = 202)	p value
age(year)	61±10.8	60±10.8	0.787
female gender	111(67.3)	89(44.1)	<0.001
medical history			
hypertention	115(69.7)	131(64.9)	0.326
diabetes mellitus	17(10.3)	33(16.3)	0.094
hyperlipemia	23(13.9)	38(18.8)	0.212
cigarette smoking	23(13.9)	60(29.7)	<0.001
heart failure	14(8.5)	30(14.9)	0.062
aspirin therapy	74(44.8)	103(51.0)	0.241
statin therapy	22(13.3)	30(14.9)	0.678
beta-blockers therapy	25(15.2)	28(13.9)	0.727
calcium channel blocker	48(29.1)	75(37.1)	0.105
ACEI/ARB therapy	42(25.5)	65(32.2)	0.159

A total of 408 patients with CAD and 367 patients without CAD were followed up for 2 to 4 years, the average follow-up time was 2.4±0.5 years. All hypertensives received regular antihypertensive drug treatment. In patients with CAD, incidence of cardiac death was 1. 5% in patients with CT and 2. 2% in patients without CT (P = 0.604). Incidence of coronary death (0.7% vs. 2.2%, P = 0.276), myocardial infarction (3.6% vs. 4. 8%, P = 0.594), as well as incidence of all cause mortality, coronary revascularization, non-fatal angina needing hospital admission and stroke were also similar between CT group and NCT group. The incidence of composite end points was also similar between the two groups (Table 6). There were no significant differences in the primary, second end point and composite end point between the CT and NCT group in fame or female with CAD (Table 7).

**Table 6:** Major adverse cardiovascular events of the patients followed-up.

MACE	CAD Patients(n = 408)			Non CAD Patients(n = 367)		
	CT group(n = 137)	NCT group(n = 271)	p value	CT group(n = 165)	NCT group(n = 202)	p value
the primary end point						
cardiac death	2(1.5)	6(2.2)	0.604	1(0.6 )	1(0.5 )	0.886
cardiac death due to CAD	1(0.7)	6(2.2)	0.276	0(0)	1(0.5)	0.365
acute myocardial infarction	5(3.6)	13(4.8)	0.594	0(0 )	2(1.0)	0.200
the secondary end point						
all cause mortality	4(2.9)	10(3.7)	0.686	2(1.2)	3(1.5)	0.822
coronary revascularization	13(9.5)	39(14.4)	0.161	1(0.6 )	1(0.5)	0.886
non-fatal angina needing hospital admission	50(36.5)	103(38.0)	0.766	1(0.6 )	1(0.5)	0.886
stroke	12(8.8)	33(12.7)	0.298	7(4.2)	9(4. 5)	0.921
composite end point	55(40.1)	130(48.0)	0.134	11(6.7)	13(6.4)	0.929

**Table 7:** Major adverse cardiovascular events of the patients with CAD followed-up.

MACE	Male (n = 249)			Female(n = 159)		
	CT group(n = 63)	NCT group(n = 186)	p value	CT group(n = 74)	NCT group(n = 85)	p value
the primary end point						
cardiac death	0(0)	3(1.6)	0.311	2(2.7 )	3(3.5 )	0.766
cardiac death due to CAD	0(0)	3(1.6)	0.311	1(1.4)	3(3.5)	0.382
acute myocardial infarction	2(3.2)	11(5.9)	0.398	3(4.1 )	2(2.4)	0.540
the secondary end point						
all cause mortality	1(1.6)	6(3.2)	0.496	3(4.1)	4(4.7)	0.842
coronary revascularization	6(9.5)	24(12.9)	0.476	7(9.5 )	15(17.6)	0.136
non-fatal angina needing hospital admission	23(36.5)	74(39.8)	0.645	27(36.5 )	29(34.1)	0.755
stroke	6(9.5)	23(12.4)	0.543	6(8.1)	10(11. 8)	0.445
composite end point	26(41.3)	89(47.8)	0.365	31( 41.9)	36(42.4)	0.953

In patients without CAD, there were no significant differences in the primary, second end point and composite end point between the two groups (Table 6).

## Discussion

The major finding of present study is that CT is positively related with essential hypertension and female gender while negatively linked with CAD in this cohort. However, prognosis may be not affected by CT in patients with CAD. CT is a common coronary angiography finding. There are several possible mechanisms responsible for the formation of CT. Artery toutuosity may be associated with age, hypertention, atherosclerosis and genetic syndrome [8]–[11]. Hemodynamic forces are vital modulators of vascular structure. Arteries may become tortuous due to reduced axial strain and hypertensive pressure in an elastic cylindrical arterial model [12]. A reduction in axial strain results in arterial tortuosity attributable to aberrant MMP activity [13]. It has been demonstrated in animal model that enlargement, elongation, and tortuosity of artery is a adaptive change to high flow and high shear stress due to smooth muscle cell proliferation and endothelial cell proliferation, and distal migration [14]. Coronary tortuosity is more pronounced in patients with chronic pressure and decreases with volume overload [5]. The close association between hypertension and CT is expected in that CT might be one of the forms of artery remodeling induced by hypertension due to increased coronary pressure and blood flow. CT could thus be recognized as an adaptive change of hypertension. Our study also found female gender is associated with higher incidence of CT compared to males. It may be due to smaller cardiac chamber of female's for coronary tortuosity decreases with cardiac enlargement [15]. The difference of CT prevalence could not be explained by the incidence of hypertension since hypertension prevalence was similar between female and male patients in this cohort.

Interestingly, our study demonstrates that CT was negatively correlated with CAD. The relationship between arterial tortuosity and atherosclerosis are customarily considered to be co-enhanced. Groves et al [16] showed that severe coronary tortuosity was associated with a significant lower incidence of significant CAD. However, tortuosity of femoral arterial was shown to enhance the development of atherosclerosis [10]. Local hemodynamic factors, especially low shear stress plays an important role in influencing atherosclerosis and plaque stability through modulating endothelial cell function and gene expression, while high shear stress is associated with protection from atherosclerosis [17]–[19]. So the negative relatiopnship between CT and CAD may due to the effect on shear stress by CT. This may be a cause for lower incidence of CAD in female.

To the best of our knowledge, this is the first study to investigate the impact of CT on prognosis of patients with CAD. Our results showed that prognosis was not affected by CT in patients with CAD. Wang et al. [20] found that the elongation and tortuosity of internal carotid artery could result in decreased blood pressure in the distal segment of tortuous internal carotid artery, kinking of internal carotid artery may be one of the factors related to attack of cerebral ischemia and there was a significant association between carotid artery kinking and transient ischemic attacks [8].

Study limitation: It is to note that the effect of artery tortuosity could be affected by hemodynamic changes such as flow pattern and shear stress, these factors were not investigated in this study and further studies are warranted to clarify related issues. Moreover, tortuous coronary arteries were not evaluated with intravascular unltrasound in our study to determine whether arterial remodeling was present in the tortuous arterial segment [16]. The relative small patient number and short follow up time might be one of the reasons for the observed neutral impact of CT on prognosis in patients with CAD. A larger cohort and longer follow up time are needed to clarify the impact of CT on prognosis of patients with CAD.

In conclusion, CT is a common angiographic finding in Chinese population with suspected CAD. CT is positively related with essential hypertension and female gender but negatively linked with CAD. However, there is no significant difference in MACE among CAD patients with or without CT. Future studies with larger patient number and longer follow up time are warranted to clarify the impact of CT on prognosis of patients with CAD. Moreover, hemodynamic changes such as coronary pressur and shear stress in CT segment should be investigated in vivo and in vitro conditions.
